# Dietary Nitrate Prevents Cardiac Dysfunction in HFrEF by Improving Hemodynamics, Ameliorating Remodeling, and Resolving Inflammation

**DOI:** 10.1111/apha.70115

**Published:** 2025-10-13

**Authors:** Miho Shimari, Gaia Picozzi, Ariela Boeder, Drielle Dantas Guimarães, Zhengbing Zhuge, Jon O. Lundberg, Mattias Carlstrom, Lars H. Lund, Daniel C. Andersson, Gianluigi Pironti

**Affiliations:** ^1^ Department of Physiology and Pharmacology Karolinska Institutet Stockholm Sweden; ^2^ Department of Molecular Biosciences, The Wenner‐Gren Institute Stockholm University Stockholm Sweden; ^3^ Cardiology Unit, Department of Medicine Karolinska Institutet Stockholm Sweden; ^4^ Cardiology Unit, Heart, Vascular and Neuro Theme Karolinska University Hospital Stockholm Sweden

## Abstract

**Aims:**

Impaired cardiac function, reduced nitric oxide (NO) bioavailability, and inflammation are key contributors to the pathogenesis and progression of heart failure with reduced ejection fraction (HFrEF). This study aimed to investigate whether dietary inorganic nitrate supplementation can attenuate cardiac dysfunction and adverse remodeling in HFrEF by enhancing NO signaling.

**Methods:**

Two mouse models of HFrEF, induced by myocardial infarction (MI) or transverse aortic constriction (TAC), were treated with dietary nitrate or a control diet for 4–6 weeks, initiating the treatment on day 3 after myocardial injury. Echocardiography and pressure volume (PV) loop analysis were employed to assess cardiac function and hemodynamics. Histology staining was performed to assess the degree of cardiac fibrosis. Myograph experiments were conducted to assess aortic vasorelaxation. Biomarkers related to hypertrophy, fibrosis, and inflammation were analyzed in cardiac tissues through Q‐PCR analysis and immunofluorescence staining.

**Results:**

In HFrEF mice, long‐term inorganic nitrate treatment increased systolic and diastolic function, enhanced vascular relaxation, and reduced both replacement and reactive fibrosis. In the nitrate group, cardiac gene expression showed downregulation of hypertrophy‐, fibrosis‐, and inflammation‐related markers, alongside upregulation of anti‐inflammatory markers associated with M1‐to‐M2 macrophage polarization. Immunofluorescence confirmed reduced fibrosis and increased anti‐inflammatory protein biomarkers associated with increased serum nitrate and cardiac cGMP levels.

**Conclusions:**

Early initiation of dietary nitrate supplementation after myocardial injury enhances cardiac and vascular function, reduces fibrosis and inflammation, and holds promise as a cardioprotective strategy to reduce the progression of HFrEF through NO‐signaling.

## 1 Introduction

Heart failure (HF) represents a life‐threatening syndrome affecting more than 64 million people globally [1] and is characterized by significant morbidity and mortality, impaired cardiac functional capacity and poor quality of life, and high costs to society [2]. The pathogenesis of heart failure involves both central and peripheral factors, with a key hallmark of acute and chronic heart failure with reduced ejection fraction (HFrEF) being the loss of contractile function. The etiology of HFrEF commonly stems from ischemic or nonischemic myocardial injury, for example, chronically elevated afterload. The heart responds to injury or stress through fibrotic processes in the form of replacement fibrosis, when cardiomyocytes are lost due to myocardial infarction, or reactive fibrosis in response to pressure overload [3]. While cardiac fibrosis occurs in response to tissue injury and is necessary for healing, excessive fibrosis can disrupt normal cardiac architecture and result in reduced contractility, increased stiffness, enhanced arrhythmogenesis, and poor outcomes [4]. Thus, myofibroblasts represent important therapeutic targets to modulate extracellular matrix accumulation in the heart.

Regardless of the underlying cause, damage to cardiac tissue triggers an inflammatory response that serves as the first step in the repair processes. Maladaptive cardiac remodeling, that occurs in the heart upon myocardial injury, is characterized by the invasion of active immune cells that secrete a wide array of pro‐inflammatory cytokines [5] including tumor necrosis factor TNF‐α, interleukin IL‐1β, and IL‐6, which are serum biomarkers associated with less favorable outcomes in HFrEF patients [6]. In the later stages of myocardial injury, the M1 macrophages polarize to M2 macrophages that enhance the healing process and the resolution of inflammatory signaling. Independently of the pathological insult, each acute inflammatory response requires a coordinated resolution process to prevent excessive inflammation and restore tissue homeostasis. Failure of this response contributes to aggravating the pathology of HF.

Nitric oxide (NO) signaling has a crucial role in cardiovascular diseases (CVD) as it influences muscle cells, angiogenesis, endothelial integrity, vascular tone, and blood pressure [7, 8, 9]. NO regulates vascular homeostasis by activating soluble guanylyl cyclase and the production of cyclic guanosine monophosphate (cGMP), which induces vascular relaxation and influences cardiac function, energy metabolism, hypertrophy, and platelet adhesion. Reduced NO bioavailability leads to decreased cGMP, resulting in increased peripheral resistance and left ventricle stiffness and fibrosis, which aggravates the pathophysiology of cardiovascular diseases [10].

Dietary inorganic nitrate (${\mathrm{NO}}_{3}^{-}$) has been shown to offer a safe and effective strategy for maintaining nitric oxide (NO) homeostasis in healthy subjects and in patients with chronic conditions [11, 12]. Through the entero‐salivary circuit, inorganic nitrate is sequentially reduced to nitrite (${\mathrm{NO}}_{2}^{-}$) and then NO [13]. However, its effects in HFrEF remain unclear, particularly regarding inotropy, hemodynamics, cardiac remodeling, and inflammation. Exploring these aspects could reveal valuable insights into its therapeutic potential in HF management.

In this study, we hypothesized that restoring NO levels through dietary inorganic nitrate could prevent HF development and progression. We investigated whether long‐term dietary nitrate supplementation could prevent HF caused by ischemic or nonischemic myocardial injury and examined physiological effects on left ventricle hemodynamics, contractility, and cardiac remodeling.

## 2 Results

### 2.1 A. Effects of Dietary Nitrate in HFrEF Induced by Myocardial Infarct (MI)

#### 2.1.1 Dietary Nitrate Enhanced Cardiac Contractility and Reduced Infarct Size

**FIGURE 1:**
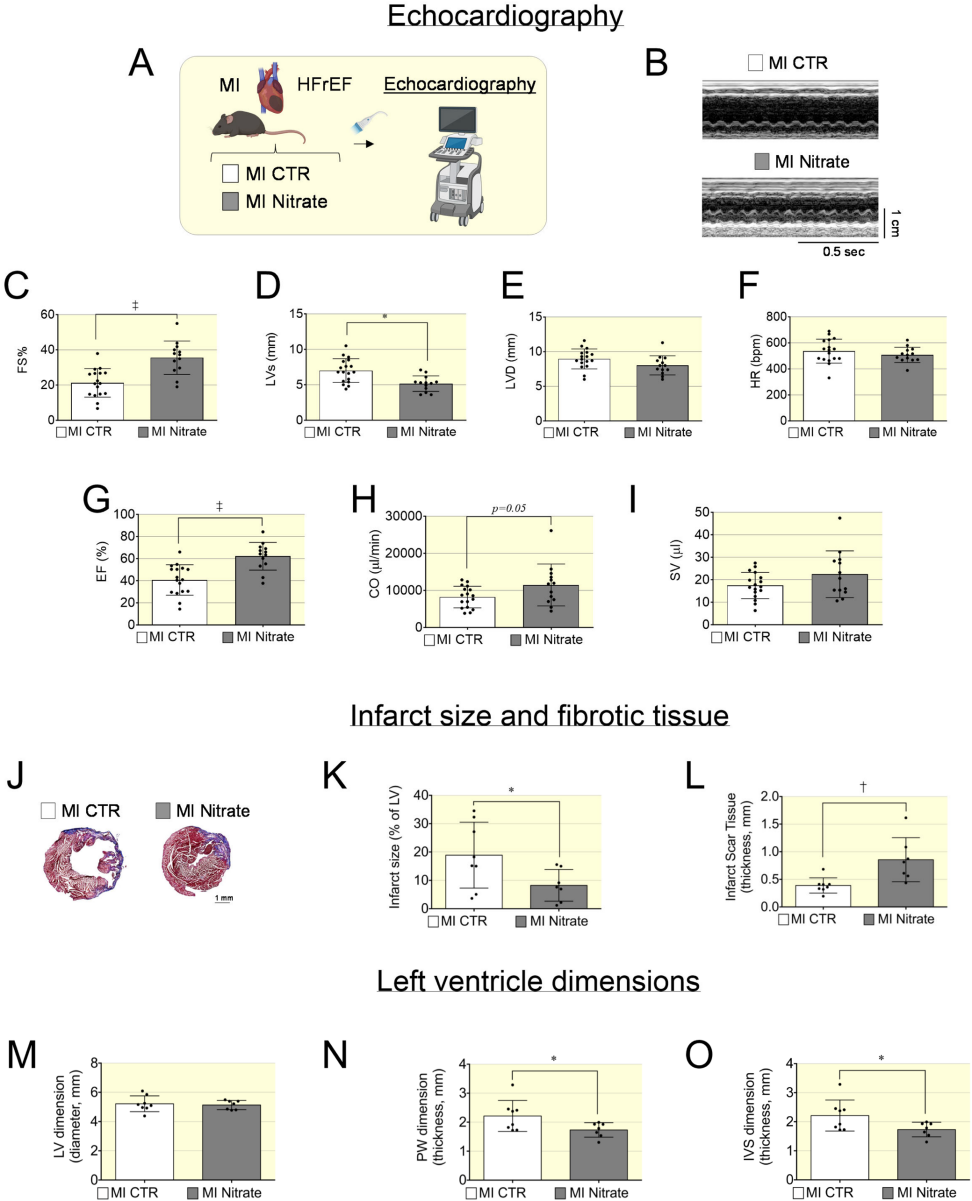
Improved contractility and reduced infarct size in hearts of mice with HFrEF post MI following dietary nitrate. (A) Cartoon depicting mice with HFrEF induced by myocardial infarct (MI) randomized for control (white box) or dietary inorganic nitrate diet (gray box) and undergoing echocardiography screening. (B) Representative M‐mode echo images of mice with HFrEF post MI from control group nitrate group. (C) Contractility of LV measured as fractional shortening (FS%) was increased in MI nitrate group compared to MI control. (D) Left ventricle systolic dimension was significantly decreased. (E) Left ventricle diastolic dimension had a trend to decrease. (F) Heart rate did not differ significantly between the control and nitrate‐treated groups. (G) Ejection fraction was significantly elevated in nitrate group while cardiac output (H) and stroke volume (I) showed a nominal increase in nitrate group. Echocardiography data presented in scatter plot with bar showing the mean and SD of mice with HFrEF post MI (*N* = 17–13). (J) Masson trichrome staining of fibrotic tissues (blue) in representative mice with HFrEF post MI hearts from nitrate and control group. (K) The infarct size was significantly reduced in nitrate group while the thickness of fibrotic scar tissue (L) was significantly increased in nitrate group. (M–O) Histological analysis of left ventricle dimensions. The left ventricle diameter (M) was not significantly different between control and nitrate group. (N) Posterior wall (PW) thickness and (O) interventricular septum (IVS) was significantly decreased in nitrate group. Data presented in scatter plot with bar showing the mean and SD of *N* = 8–7 samples. Unpaired *t* test was used to compare two groups (HFrEF post MI Control and HFrEF post MI Nitrate groups). A *p* < 0.05 was considered statistically significant where * indicates *p* < 0.05, † *p* < 0.01 and ‡ *p* < 0.001.

Mice with HF induced by MI were treated at day 3 post‐surgery with an inorganic nitrate enriched diet or control diet for 4–6 weeks. Echocardiography screening was performed to assess cardiac function after treatment (Figure 1A,B). Mice in the nitrate group showed a significant increase in contractility (fractional shortening FS%) compared to the control group (Figure 1C). Left ventricular systolic dimension (LVs) was significantly reduced and the diastolic dimension (LVD) showed a trend toward being lower in the nitrate group (Figure 1D,E). Ejection fraction was significantly elevated while cardiac output and stroke volume were nominally elevated in the nitrate group. Heart rate was not significantly different between groups (Figure 1F–I).

Beyond its effect on contractile function, nitrate treatment reduced the infarct size and fibrotic scar tissue formation (evident by blue staining in the Masson staining) following MI (Figure 1J,K). The fibrotic scar tissue appeared to be thicker in the nitrate group compared to the control group (Figure 1L). In fact, treatment with inorganic nitrate effectively confined ischemic injury to a smaller, more localized area, whereas the extent of scar tissue damage was significantly larger in the heart of HFrEF mice that received a control diet. Thus, an inorganic nitrate‐enriched diet limits the advancement of myocardial injury during HF, emphasizing its potential as a preventive therapy against adverse cardiac remodeling. Histological analysis showed that the dimension of LV diameter was not significantly changed between the nitrate and control groups (Figure 1M). However, the thickness of the interventricular septum (IVC) and posterior wall (PW) was significantly decreased in the hearts of MI mice treated with an inorganic nitrate‐enriched diet (Figure 1N,O).

#### 2.1.2 Dietary Nitrate Decreased Biomarkers of Wall Stress/Hypertrophy and Fibrosis in Failing Hearts

**FIGURE 2:**
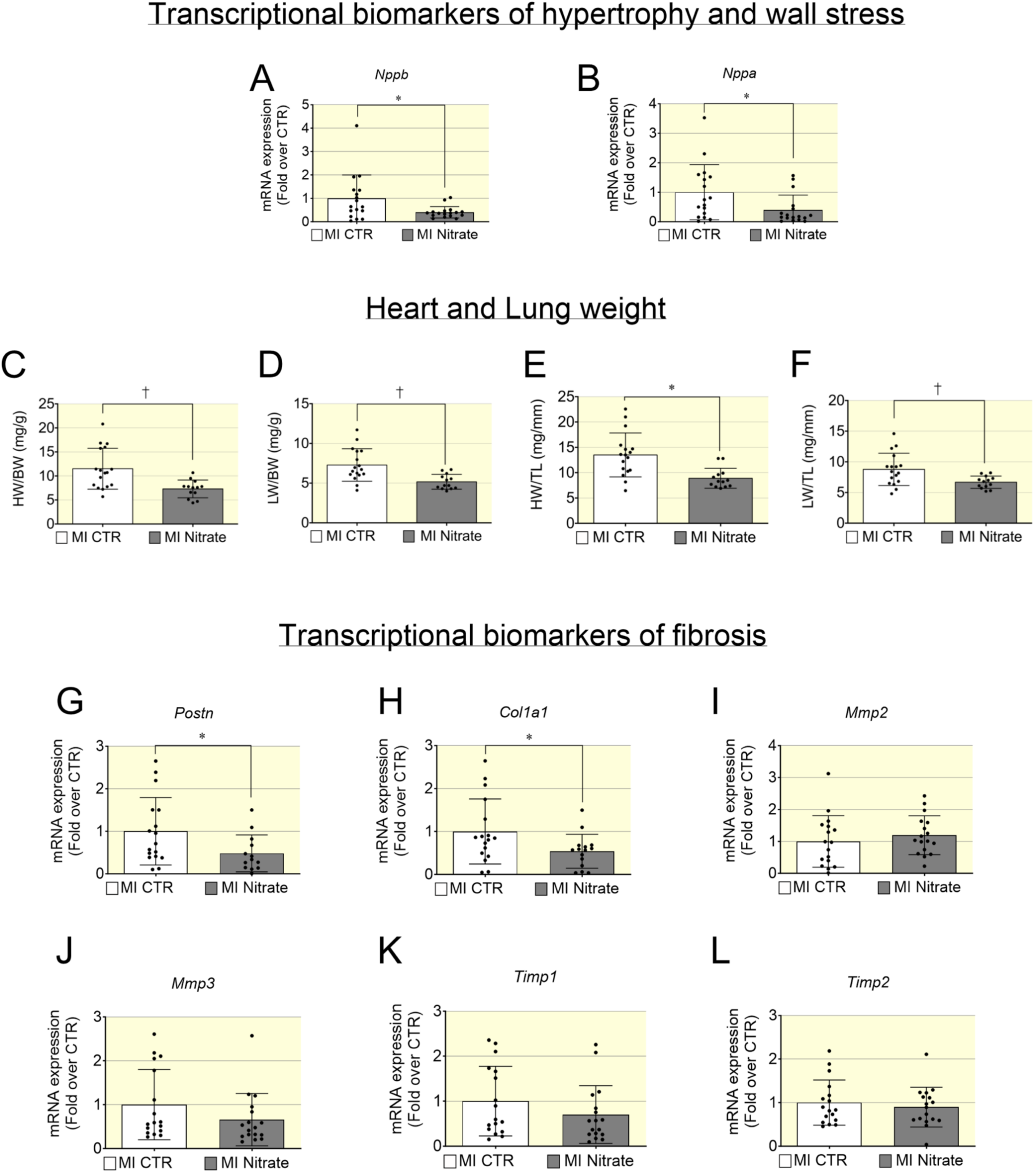
Decreased hypertrophy biomarkers, pulmonary congestion, and cardiac fibrosis in HFrEF post MI following inorganic nitrate‐enriched diet. (A, B) In mice with HFrEF post MI treated with inorganic nitrate enriched diet, the mRNA levels of genes linked to hypertrophy/wall stress such as brain natriuretic peptide (*Nppb*) and atrial natriuretic peptide (*Nppa*) were significantly decreased compared to control group. (C) The heart weight to body weight ratio was used as surrogate of cardiac hypertrophy and was significantly decreased in nitrate group. (D) Lung weight to body weight ratio was used as surrogate of pulmonary congestion and this was significantly decreased in nitrate group. Heart weight and Lung weight were also normalized to tibial length (E, F) to account for potential differences in body size and ensure a more accurate comparison between groups. (G, H) Transcriptional level of profibrotic genes such as periostin (*Postn*) (G) and collagen I (*Col1a1*) (H) were significantly decreased in nitrate group. The mRNA levels of extracellular turnover biomarkers such as metalloproteases *Mmp2* (I), *Mmp3* (J), and tissue inhibitors of metalloproteases *Timp1* (K) and *Timp2* (L) were not significantly changed between nitrate and control group. Data presented in scatter plot with bar showing the mean and SD of *N* = 17–13 hearts analyzed. Unpaired *t* test was used to compare two groups (HFrEF post MI Control and HFrEF post MI Nitrate groups). A *p* < 0.05 was considered statistically significant where * indicates *p* < 0.05 and † *p* < 0.01.

Analysis of transcriptional biomarkers in cardiac tissues obtained from mice with HFrEF induced by MI showed that nitrate treatment reduced the levels of transcriptional biomarkers of cardiac remodeling including wall stress/hypertrophy such as *Nppb* and *Nppa* (Figure 2A,B). Moreover, the heart weight and the lung weight, normalized to either body weight (Figure 2C,D) or tibial length (Figure 2E,F), were significantly decreased in the nitrate group. These results are consistent with a decreased wall stress/hypertrophy of the heart and decreased pulmonary congestion in HFrEF mice treated with an inorganic nitrate‐enriched diet.

Cardiac tissues obtained from mice with HFrEF post MI, treated with an inorganic nitrate enriched diet, showed a significant reduction of transcriptional cardiac fibrosis biomarkers involved in extracellular matrix deposition such as *Postn* and *Col1a1* (Figure 2G,H). There were no statistical differences between groups in mRNA levels of biomarkers linked to extracellular matrix (ECM) turnover such as metalloproteases *Mmp2*, *Mmp3* (Figure 2I,J) and tissue inhibitor of metalloproteases such as *Timp1*, *Timp2* (Figure 2K,L). These results indicate that inorganic nitrate treatment modulates ECM deposition rather than turnover in cardiac tissues of HFrEF post MI mice.

#### 2.1.3 Dietary Nitrate Reduced Pro‐Inflammatory and Increased Anti‐Inflammatory Biomarkers

**FIGURE 3:**
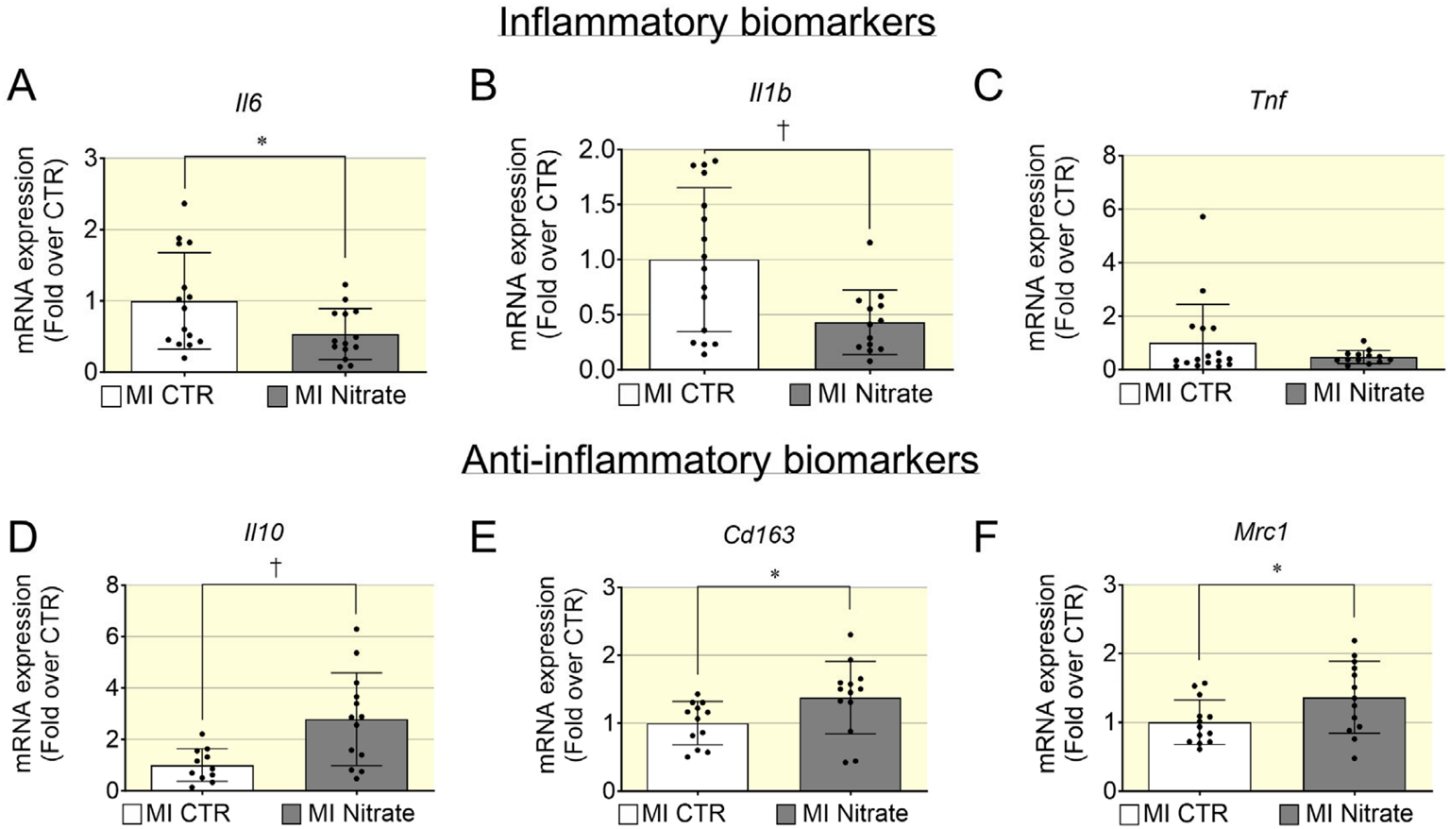
Pro‐inflammatory and anti‐inflammatory transcriptional biomarkers in HFrEF post‐MI following inorganic nitrate‐enriched diet. (A, B) The mRNA levels of pro‐inflammatory biomarker such as *Il6* and *Il1b* (B) were significantly decreased in nitrate group. (C) There were no significant differences in *Tnf* mRNA levels between the control and nitrate groups, although the MI control group exhibited a broader distribution of expression values. (D–F) Anti‐inflammatory cytokines such as *Il10* (D) and biomarkers of anti‐inflammatory macrophages M2 for example *Cd163* (E) and *Mrc1* (F) were significantly increased in hearts of mice with HFrEF post MI treated with nitrate. Data presented in scatter plot with bar showing the mean and SD of *N* = 17–13 samples. Unpaired *t* test was used to compare control and nitrate groups. A *p* < 0.05 was considered statistically significant where * indicates *p* < 0.05 and † *p* < 0.01.

We analyzed the transcriptomic profile of inflammatory and anti‐inflammatory biomarkers in cardiac tissues of mice with HFrEF post MI and observed that dietary inorganic nitrate treatment was associated with a significant reduction of pro‐inflammatory cytokines such as *Il6* and *Il1b* (Figure 3A,B). *Tnf* levels showed a larger spread in the control group; however, there were no significant differences between control and nitrate groups (Figure 3C). Nevertheless, the levels of anti‐inflammatory biomarkers such as *Il10* and biomarkers of M2 macrophages such as *Cd136* and *Mrc1* (coding for mannose receptor, C type 1, known also as CD 206) were significantly increased in the nitrate group (Figure 3D–F). Thus, an inorganic nitrate enriched diet in HF post MI enhanced the anti‐inflammatory profile in the heart through M1 to M2 macrophage polarization, which might be linked to the blunted fibrotic response.

#### 2.1.4 Increased Anti‐Inflammatory and Decreased Fibrosis Biomarkers Are Associated With Elevated Serum Nitrate and Cardiac cGMP Levels in HFrEF Post‐MI Mice

**FIGURE 4:**
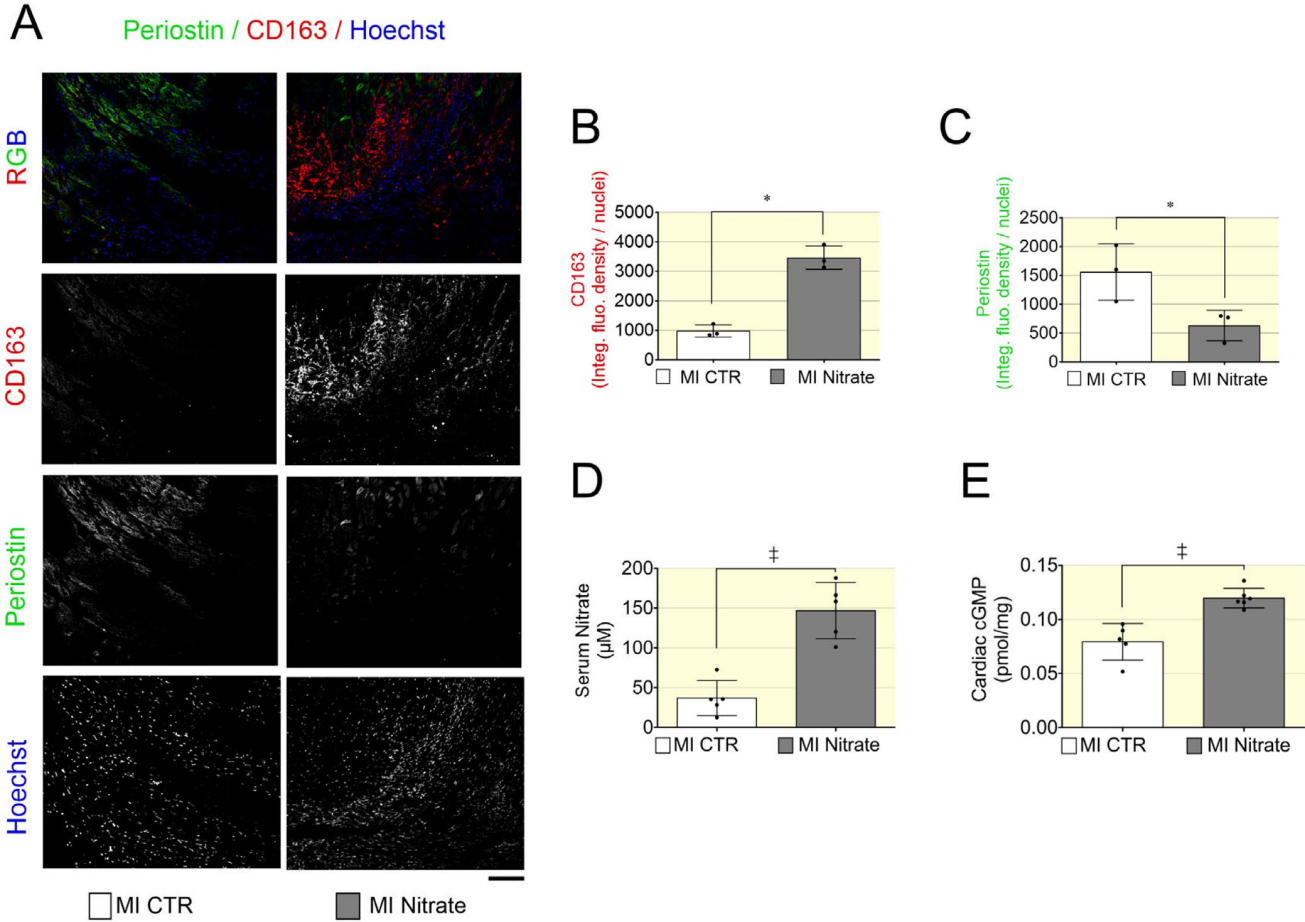
Increased expression of anti‐inflammatory and reduced fibrosis‐related markers is associated with elevated serum nitrate and cGMP levels in the heart. (A) Representative immunofluorescence staining of cardiac tissue showing Periostin, CD163, and nuclear counterstaining with Hoechst, as indicated. Scale bar indicating 100 μm. Immunofluorescence signal was expressed as integrated fluorescence density normalized to nuclei per field and showed increased expression levels of CD163 (B) and decreased Periostin (C) in nitrate treated group. Data presented in scatter plot with bar showing the mean and SD of *N* = 3 mice used to collect an equal number of images randomly selected from matched anatomical regions of the left ventricle at 20× magnification. Kruskal–Wallis nonparametric test was used to compare control and nitrate groups. (D) Serum nitrate levels in MI mice receiving either control or nitrate enriched diet. (E) cGMP concentration measured in cardiac tissues. Data in (D and E) are shown in scatter plot with bar showing the mean and SD of *N* = 5–6 mice with HFrEF post MI as indicated. A *p* < 0.05 was considered statistically significant where * indicates *p* < 0.05 and ‡ *p* < 0.001.

Cardiac cryosection of MI mice was stained for CD163, Periostin, and nuclear counterstaining with Hoechst, as indicated (Figure 4A). Hearts from the nitrate‐treated group exhibited increased CD163 expression and reduced Periostin levels (Figure 4B,C), suggesting that dietary nitrate promotes M2 macrophage polarization while suppressing myofibroblast activation in the hearts of HFrEF mice. These findings align with the anti‐inflammatory and anti‐fibrotic effects of dietary nitrate in heart failure. Notably, mice receiving the nitrate‐enriched diet also displayed significantly elevated serum nitrate levels and increased cardiac cGMP concentrations (Figure 4D,E), supporting the involvement of the nitrate–nitrite‐NO pathway and activation of sGC‐cGMP signaling in mediating the observed cardiac anti‐inflammatory and anti‐fibrotic responses.

### 2.2 Dietary Nitrate Effects in HFrEF Induced by Transverse Aortic Constriction (TAC)

#### 2.2.1 Dietary Nitrate Supplementation Improved Hemodynamics and Cardiac Function

To evaluate the impact of an inorganic nitrate‐enriched diet on HF driven by nonischemic cardiomyopathy (e.g., pressure overload), we employed a well‐established model of HF induced by transverse aortic constriction (TAC) in mice [14, 15]. This model mimics the pathophysiology of human HF due to pressure overload, and it is a more suitable model for Pressure‐Volume (PV) loop experiments compared to the MI model, from a technical standpoint, given the risk of ventricular rupture and nonsurvival outcomes. Although both the MI and TAC models exhibit impaired systolic function and are referred to as the HFrEF model, the degree of dysfunction was more pronounced in MI, as indicated by a significant reduction in FS% and EF% compared to TAC (Figure S1).

**FIGURE 5:**
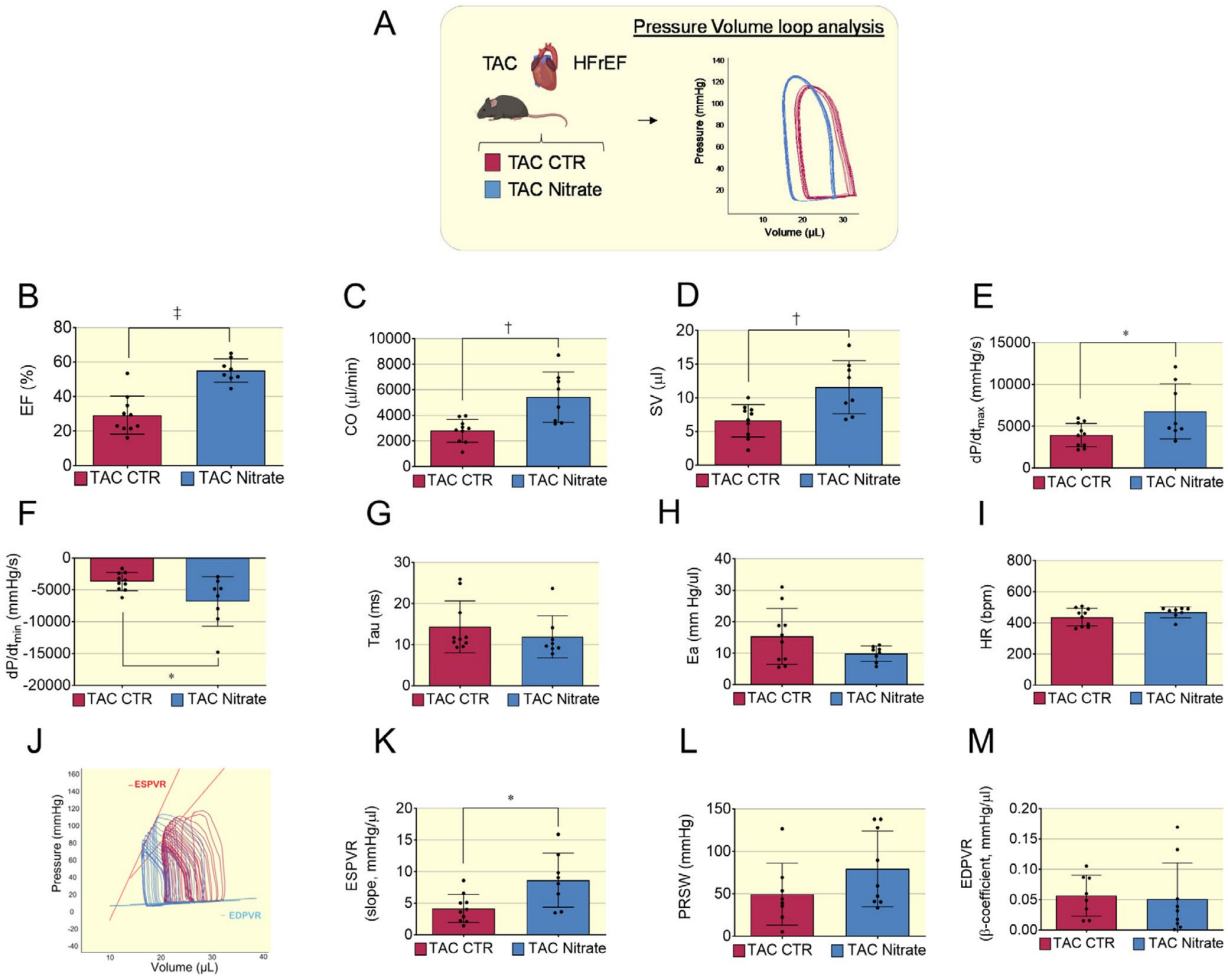
Improved hemodynamics in HFrEF induced by TAC following dietary nitrate. (A) Cartoon depicting mice with HFrEF induced by transverse aortic constriction (TAC) randomized for control (red box) or dietary nitrate (blue box) diet and undergoing Pressure volume (PV) loop analysis. Representative PV loops traces showing nitrate group shifted to the left. (B–D) Mice with HFrEF induced by TAC treated with nitrate showed a significant increased ejection fraction (EF%), Cardiac output (CO), and Stroke volume (SV). (E) The rate of maximal left ventricle pressure development (dP/dtmax) was significantly increased in nitrate group. (F) The rate of minimum pressure development (dP/dtmin) was significantly decreased. (G) Time of pressure development decay and (H) Arterial Elastance (Ea) was slightly decreased in TAC nitrate group compared to TAC control indicating an improvement of myocardial compliance to LV blood filling. (I) Heart rate did not show significant difference between control and nitrate‐treated groups. Data presented in scatter plot with bar showing the mean and SD of *N* = 10–8 mice with HFrEF post TAC as indicated. (J–M) Increased load independent systolic function. Load independent systolic and diastolic parameters were obtained following inferior vena cava (IVC) occlusion. (J) Representative PV loops of mice HFrEF post TAC in control (red) or nitrate (blue) groups obtained upon IVC occlusion. End systolic pressure volume relationship (ESPVR) curve is in red and end diastolic pressure volume relationship (EDPVR) curve is cyan. (K) ESPVR slope was significantly increased in nitrate group. (L) preload‐recruitable stroke work (PRSW) tends to increase in nitrate group. (M) No significant changes between groups were observed in end diastolic pressure volume relationship (EDPVR) curves. Data presented in scatter plot with bar showing the mean and SD of *N* = 10–8 mice with HFrEF post TAC as indicated. Unpaired *t* test was used to compare control and nitrate groups. A *p* < 0.05 was considered statistically significant where * indicates *p* < 0.05, † *p* < 0.01 and ‡ *p* < 0.001.

Invasive PV loop analysis revealed that 4–6 weeks of chronic treatment with an inorganic nitrate‐enriched diet induced a leftward shift of the loop (Figure 5A) and significantly improved hemodynamics in the failing hearts by enhancing both systolic and diastolic function compared to controls. HFrEF mice in the nitrate group presented a significant improvement in systolic function with increased ejection fraction, cardiac output, stroke volume, and rate of maximal pressure development (dP/dt max) (Figure 5A–E). The rate of minimum pressure development (dP/dt min) was significantly reduced (Figure 5F), indicating that diastolic function was improved, while the time of relaxation (Tau) and myocardial stiffness (elastance, Ea) showed a trend to decrease in the nitrate group (Figure 5G,H). There was no significant difference in heart rate between the control and nitrate‐treated groups (Figure 5I).

Load independent parameters obtained upon inferior vena cava (IVC) occlusion showed that inorganic nitrate treatment had a significant effect on contractility (Figure 5J). In fact, the slope of the end systolic pressure volume relationship (ESPVR) curve was significantly increased and the preload recruitable stroke work (PRSW) was nominally increased (Figure 5K,L). However, load independent diastolic function, assessed by end diastolic pressure volume relationship (EDPVR) curve, was not significantly different between the control and nitrate groups (Figure 5M).

Moreover, cardiac function was assessed noninvasively through echocardiography analysis (Figure S2A). Similar to what was seen in the PV loop study, dietary nitrate treatment significantly increased left ventricular fractional shortening (Figure S2B). The left ventricle diastolic and systolic dimensions (LVD and LVs) were significantly decreased (Figure S2C,D). There was a trend to decrease in the thickness of the interventricular septum (IVS) and a neutral effect on posterior wall (PW) thickness (Figure S1E,F). Heart rate (HR) showed a small trend to increase with nitrate treatment, and ejection fraction and cardiac output were significantly elevated in the nitrate group, while there was a neutral effect on stroke volume of mice with HFrEF post TAC (Figure S1G–J).

#### 2.2.2 Improved Vascular Relaxation Following Dietary Nitrate Supplementation

**FIGURE 6:**
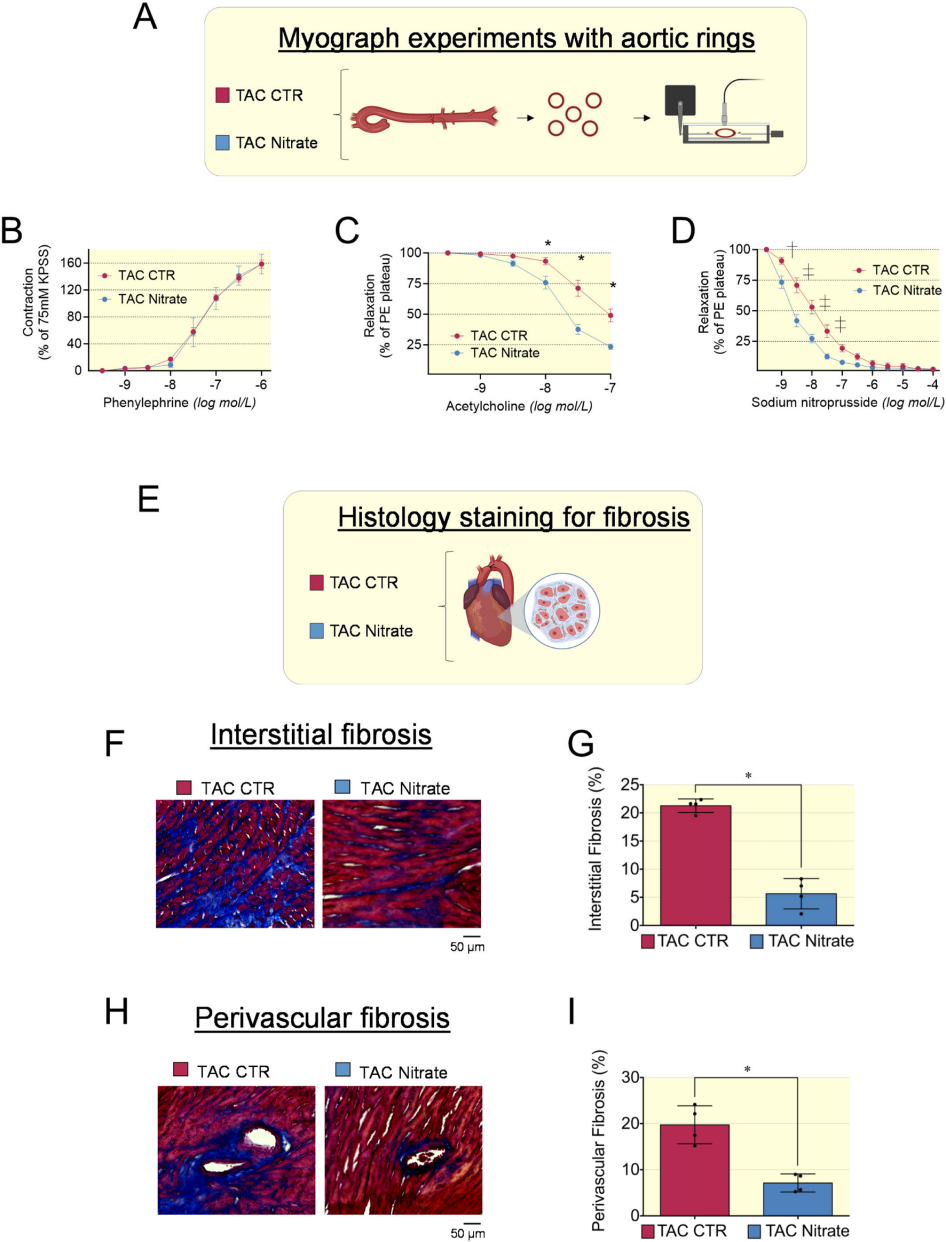
Enhanced relaxation of aortic rings and reduced cardiac fibrosis. (A) Cartoon depicting myograph experiments with aortic rings isolated from mice with HFrEF induced by TAC and randomized for control (red box) or nitrate diet (blue cox). (B) Contractility of aortic rings, assessed through stimulation with phenylephrine, was no different between nitrate and control groups. (C) Endothelial dependent relaxation assessed through stimulation with acetylcholine. (D) Endothelial independent relaxation assessed through stimulation with sodium nitroprusside. Both endothelial dependent and endothelial independent relaxation function were significantly improved in nitrate group. Aortic rings obtained from 3 HFrEF mice in each group. Data presented in scatter plot with bar showing the mean and SD. Repeated two‐way ANOVA followed by post hoc test was used to compare vascular responses in the control and nitrate groups. A *p* < 0.05 was considered statistically significant where * indicates *p* < 0.05, † *p* < 0.01 and ‡ *p* < 0.001. (E–I) Reduced cardiac fibrosis in hearts of HFrEF mice induced by TAC treated with inorganic nitrate enriched diet. (E) Cartoon depicting histology staining for fibrosis in hearts of mice with HFrEF induced by TAC and randomized for control or nitrate diet. (F) Representative Masson trichrome staining of interstitial fibrosis and (G) mean ± SD showing a significant reduction in nitrate group. (H) Representative Masson trichrome staining of perivascular fibrosis and (I) mean and SD showing a significant reduction in nitrate group. Data presented in scatter plot with bar of *N* = 4 mice used to collect an equal number of images randomly selected from matched anatomical regions of interstitial and perivascular areas at 20× magnification. Kruskal–Wallis nonparametric test was used to compare control and nitrate groups. A *p* < 0.05 was considered statistically significant where * indicates *p* < 0.05.

Myograph experiments were performed on aortic vessels isolated from mice with HFrEF induced by TAC (Figure 6A). Phenylephrine caused concentration‐dependent contraction in a similar manner between nitrate and control (Figure 6B). However, treatment with an inorganic nitrate‐enriched diet caused a significant leftward shift of the relaxation of aortic rings isolated from HFrEF induced by TAC, as reported in concentration–response curves (Figure 6C,D). In fact, both vasorelaxations, endothelium‐dependent (induced by acetylcholine, Ach) and endothelium‐independent (induced by sodium nitroprusside, SNP), were improved in mice treated with dietary nitrate (Figure 6C,D).

The vascular relaxation effects of an inorganic nitrate‐enriched diet align with the hemodynamic improvements seen in HFrEF mice, likely contributing to the overall enhancement of both systolic and diastolic cardiac function.

#### 2.2.3 Reduced Cardiac Interstitial and Perivascular Fibrosis Following Dietary Nitrate Supplementation

Histology staining for cardiac fibrosis in hearts of mice with HFrEF induced by TAC (Figure 6E) showed that cardiac fibrosis, both interstitial and perivascular, was significantly reduced in the nitrate group compared to TAC controls (Figure 6F–I). The reduction in fibrosis may account for the observed decrease in myocardial stiffness (Ea) and improved diastolic function. However, the left ventricular loading conditions likely play a critical role in lowering wall stress and contributing to the overall beneficial effects on diastolic function of dietary nitrate treatment.

#### 2.2.4 Decreased Wall Stress and Hypertrophy After Dietary Nitrate Supplementation

**FIGURE 7:**
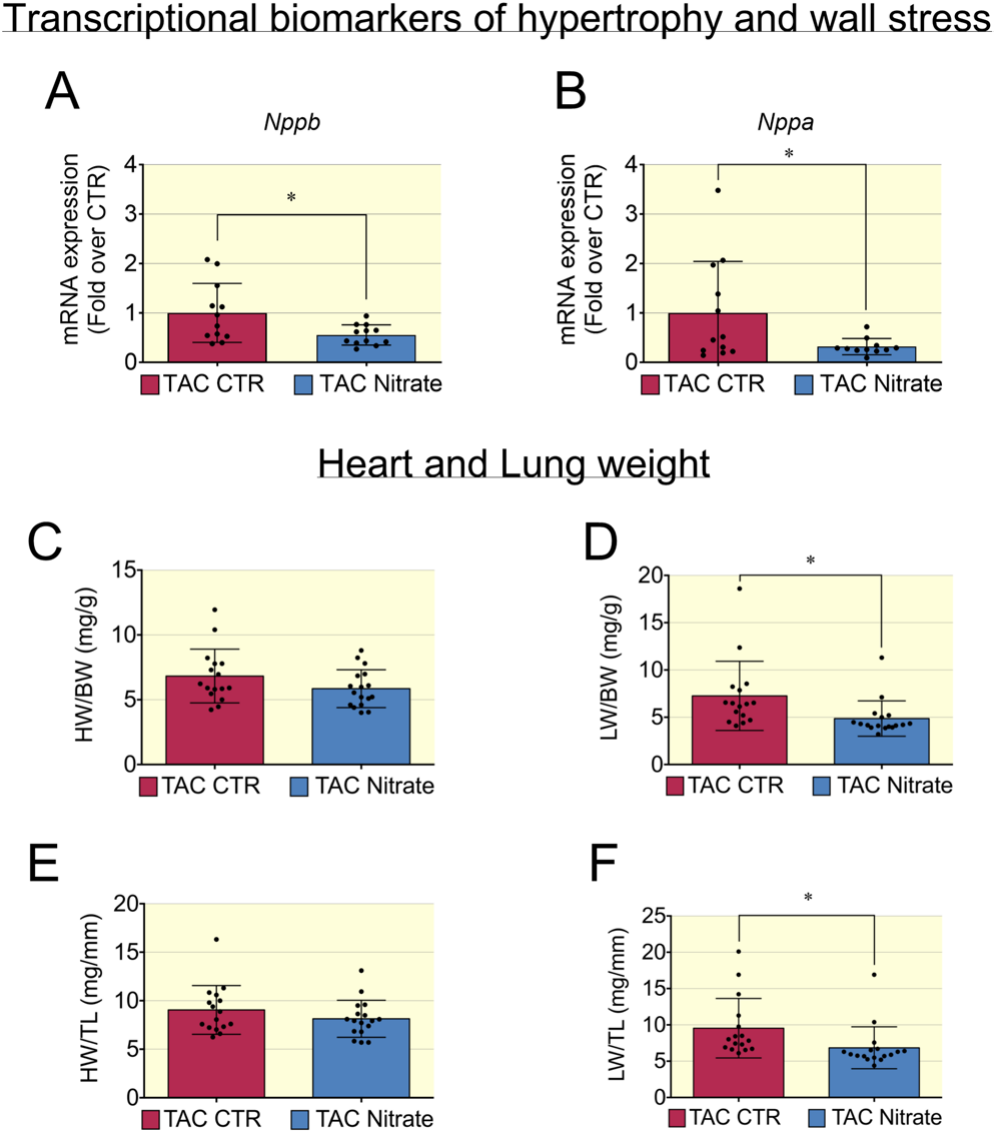
Decreased levels of biomarkers for wall stress/hypertrophy and pulmonary congestion in HFrEF post TAC. (A, B) In mice with HFrEF post TAC treated with inorganic nitrate enriched diet, the mRNA levels of genes linked to wall stress/hypertrophy such as brain natriuretic peptide (*Nppb*) and atrial natriuretic peptide (*Nppa*) were significantly decreased compared to control group. Data presented in scatter plot with bar showing the mean and SD of *N* = 15–12 samples in Q‐PCR experiments. (C) The heart weight to body weight ratio (HW/BW) was used as surrogate of cardiac hypertrophy and was significantly decreased in nitrate group. (D) Lungs weight to body weight ratio (LW/BW) was used as surrogate of pulmonary congestion and this was significantly decreased in nitrate group. Heart weight and lung weight was also normalized to tibial length (E, F) to account for potential differences in body size and ensure a more accurate comparison between groups. Data presented in scatter plot with bar showing the mean and SD of *N* = 16–17 mice with HFrEF post TAC. Unpaired *t* test was used to compare two groups (HFrEF post TAC Control and HFrEF post TAC Nitrate groups). A *p* < 0.05 was considered statistically significant where * indicates *p* < 0.05.

Analysis of transcriptional biomarkers in hearts of mice with HFrEF induced by the TAC model showed that inorganic nitrate treatment reduced the mRNA levels of genes involved in wall stress/hypertrophy such as *Nppb* and *Nppa* (Figure 7A,B). Heart weight, normalized to either body weight or tibial length, was used as a surrogate of cardiac hypertrophy and showed only a trend to decrease in the nitrate group (Figure 7C–E). Pulmonary congestion was significantly improved since lung weight was significantly decreased in the nitrate group (Figure 7D–F).

#### 2.2.5 Decreased Fibrosis Biomarkers After Dietary Nitrate Supplementation

**FIGURE 8:**
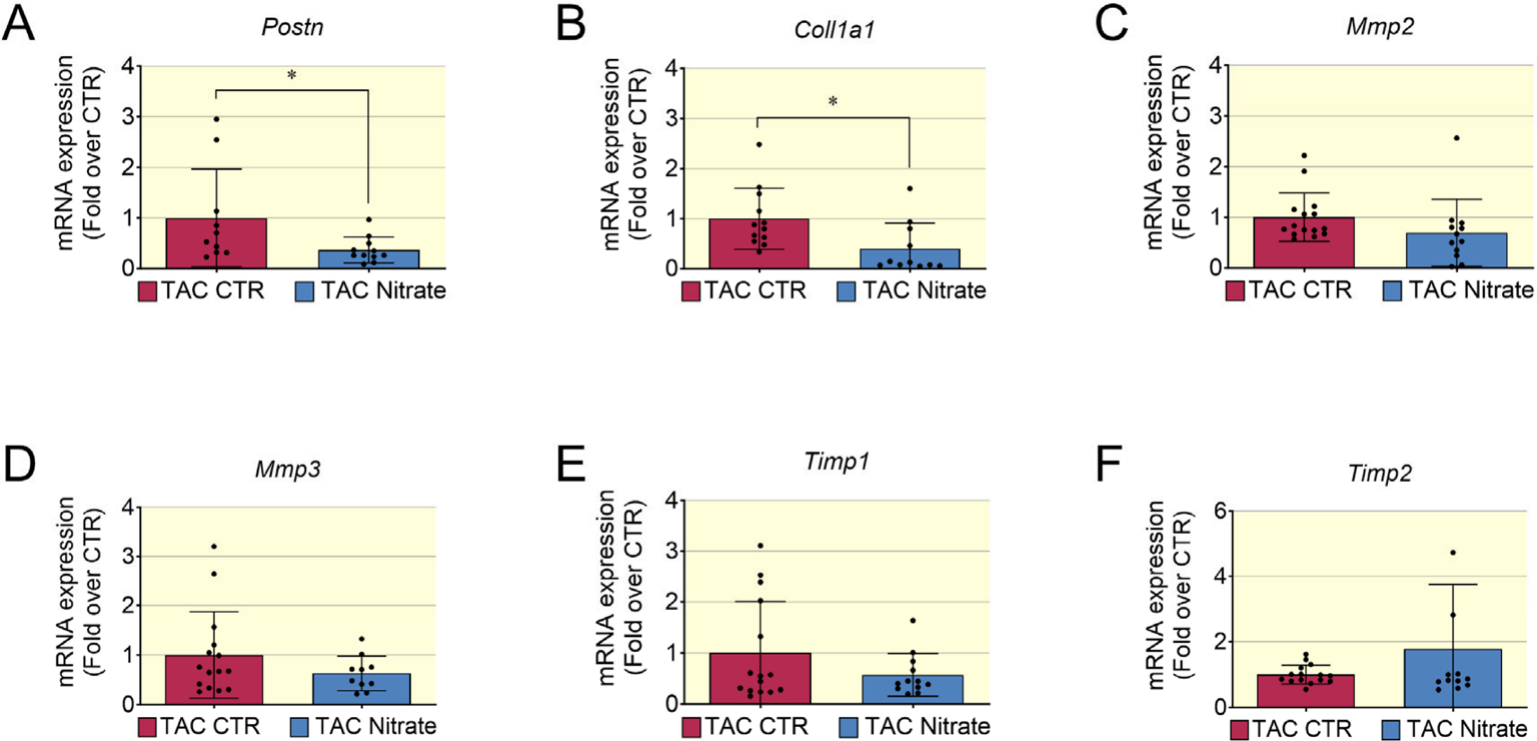
Transcriptional biomarkers of cardiac fibrosis in HFrEF post TAC. (A, B) Transcriptional level of genes involved in extracellular matrix deposition such as *Postn* and *Col1a1* were significantly decreased in nitrate group. (C) The transcriptional levels of metalloproteases *Mmp2* and *Mmp3* (D) were not significantly different between control and nitrate group. (E, F) There was no statistically significant change in mRNA levels of extracellular turnover biomarkers such as, *Timp1* (E) and *Timp2* (F) between nitrate and control group. Data presented in scatter plot with bar showing the mean and SD of *N* = 15–12 samples analyzed. Unpaired *t* test was used to compare two groups (HFrEF post TAC Control and HFrEF post TAC Nitrate groups). A *p* < 0.05 was considered statistically significant where * indicates *p* < 0.05.

Cardiac fibrosis biomarkers such as *Postn* and *Col1a1* were significantly decreased (Figure 8A,B). The transcriptional levels of metalloproteases involved in the extracellular matrix degradation, such as *Mmp2* and *Mmp3*, were not significantly changed between control and nitrate groups (Figure 8C,D). There were no significant changes also in mRNA levels of genes involved in the modulation of extracellular matrix turnover such as tissue inhibitors of metalloproteases *Timp‐1* and *Timp‐2* (Figure 8E,F). These results are consistent with findings obtained from the MI model where 4–6 weeks of treatment with an inorganic nitrate‐enriched diet in HFrEF mice significantly decreased the transcriptional levels of genes involved in ECM deposition rather than proteins involved in ECM turnover.

#### 2.2.6 Reduced Pro‐Inflammatory and Increased Anti‐Inflammatory Biomarkers Following Dietary Nitrate Supplementation

**FIGURE 9:**
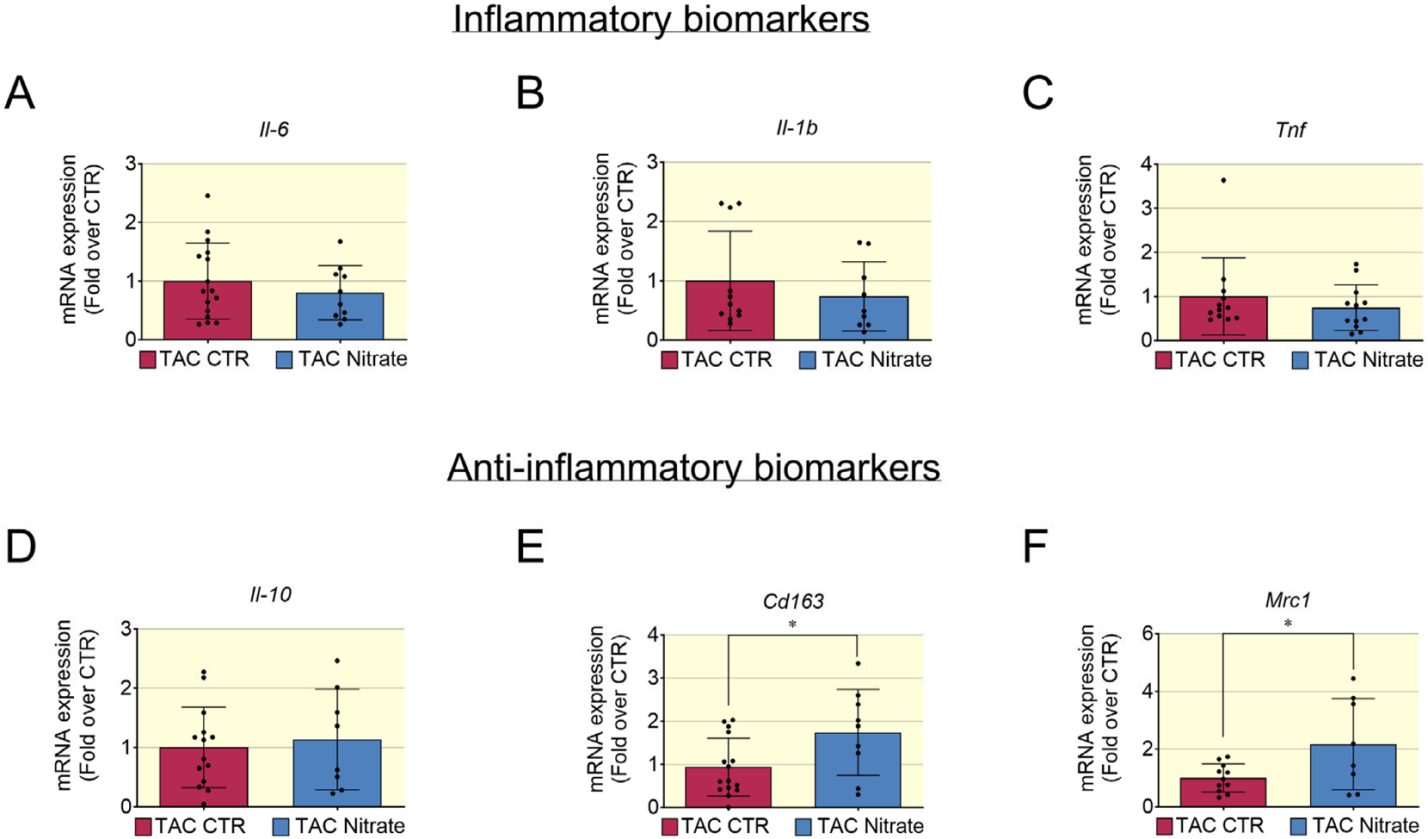
Pro‐inflammatory and anti‐inflammatory biomarkers in HFrEF post TAC. (A–C) The mRNA levels of pro‐inflammatory biomarkers (*Il6, Il1b* and *Tnf*) was not significantly changed between control and nitrate group. The HFrEF post TAC control group showed a tendency to increase proinflammatory biomarkers given to a few samples that appeared to have higher levels of pro‐inflammatory cytokines. (D) The transcriptional levels of anti‐inflammatory cytokines *Il10* was not significantly changed between control and nitrate group. (E) The mRNA level of biomarkers of anti‐inflammatory macrophages M2 for example *Cd163* and *Mrc1* (F) were significantly increased in hearts of mice with HFrEF post TAC treated with inorganic nitrate diet. Data presented in scatter plot with bar showing the mean and SD of *N* = 14–8 samples analyzed. Unpaired *t* test was used to compare two groups (HFrEF post TAC Control and HFrEF post TAC Nitrate groups). A *p* < 0.05 was considered statistically significant where * indicates *p* < 0.05.

We analyzed the transcriptional profile of inflammatory and anti‐inflammatory biomarkers in cardiac tissues of mice with HFrEF induced by TAC. Inorganic nitrate enriched diet treatment had a neutral effect on transcriptional levels of pro‐inflammatory cytokines such as *Il6*, *Il1b*, and *Tnf; however, the* TAC control group displayed a few samples with higher levels of pro‐inflammatory cytokines (Figure 9A–C). Furthermore, the inorganic nitrate enriched diet appeared to have a neutral effect on transcriptional levels of the cytokine *Il10*, while the statistically significant increase of *Cd163* and *Mrc1* levels suggests a more prominent M2 phenotype induced by dietary nitrate (Figure 9D–F).

#### 2.2.7 Increased Expression of M2 Macrophages Biomarkers Is Associated With Elevated Serum Nitrate and Cardiac cGMP Levels in HFrEF Post‐TAC Mice

**FIGURE 10:**
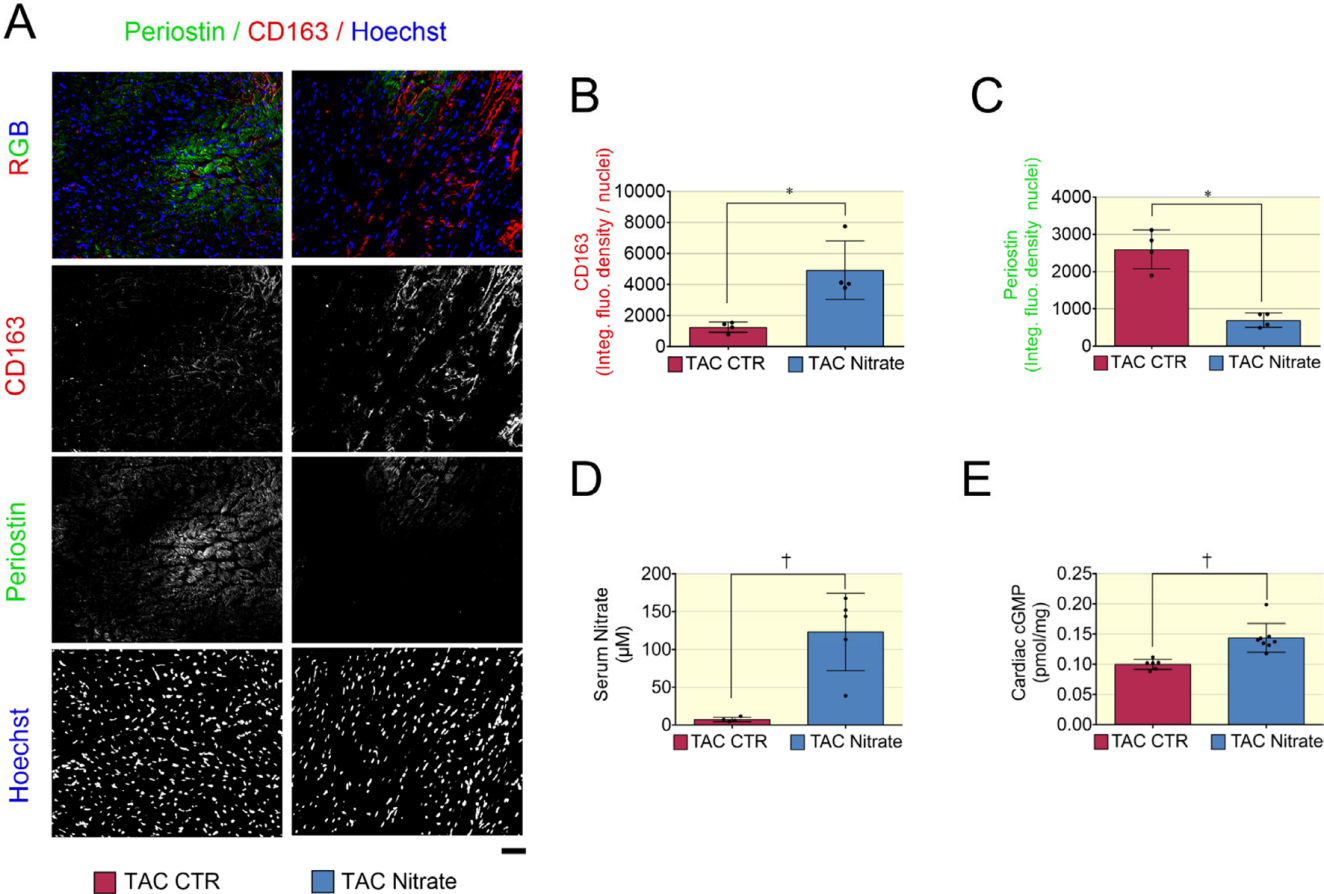
Increased M2 macrophages and reduced activated myofibroblasts related markers are associated with elevated serum nitrate and cardiac cGMP levels in the heart of TAC mice. (A) Representative immunofluorescence staining of cardiac tissue showing CD163, Periostin, and nuclear counterstaining with Hoechst, as indicated. Scale bar indicating 50 μm. Quantification was expressed as integrated fluorescence density normalized to nuclei per field and showed increased expression levels of CD163 (B) and decreased Periostin (C) in the nitrate treated group. Data presented in a scatter plot with a bar showing the mean and SD of *N* = 4 mice used to collect an equal number of images randomly selected from matched anatomical regions of the left ventricle at 20× magnification. Kruskal–Wallis nonparametric. An unpaired *t* test was used to compare control and nitrate groups. (D) Serum nitrate levels in TAC mice receiving either a control or nitrate enriched diet. (E) cGMP concentration measured in cardiac tissues. Data in D and E are shown in box and whiskers graphs as mean ± SD of *N* = 5–6 mice with HFrEF post TAC as indicated. A *p* < 0.05 was considered statistically significant where * indicates *p* < 0.05 and † *p* < 0.01.

Hearts from the nitrate‐treated TAC mice exhibited increased CD163 expression and reduced Periostin levels compared to the control group (Figure 10A–C). Additionally, serum nitrate levels and cardiac cGMP concentrations were significantly increased in the nitrate group (Figure 10D,E). These findings further support the role of the nitrate–nitrite–NO pathway and subsequent sGC–cGMP signaling in mediating anti‐inflammatory and anti‐fibrotic effects in nonischemic HFrEF.

## 3 Discussion

Heart failure with reduced ejection fraction is characterized by a significant decline in systolic function, often observed as a reduced cardiac output. This decline is closely tied to pathological cardiac remodeling, including fibrosis, and accompanying vascular dysfunction, both of which exacerbate the progression of HF in a vicious circle and hinder recovery. Modulation of the underlying pathophysiological mechanisms is an unmet need in terms of both preventive and therapeutic strategies. We conducted a comprehensive study using two animal models of HFrEF induced by either MI or TAC. We showed that in mice with HFrEF, an inorganic nitrate enriched diet compared to the control diet (i) improves left ventricle systolic function in both load‐dependent and independent manners, (ii) improves vascular relaxation and enhances diastolic function, (iii) prevents the aggravation of cardiac remodeling by reducing replacement fibrosis in HFrEF post‐MI and reactive fibrosis in HFrEF post‐TAC, and (iv) increases the expression of anti‐inflammatory and decreases pro‐inflammatory biomarkers in failing hearts. Thus, the cardioprotective effects of inorganic nitrate enriched diet treatment were associated with the M1 to M2 macrophage polarization, which is a critical step to promote the resolution of inflammatory signaling [16, 17].

### 3.1 Clinical Relevance of Nitrate–Nitrite–NO Signaling

Our findings are based exclusively on studies conducted in male mice and therefore have limited generalizability to females. Previous research suggests that females may exhibit a less pronounced physiological response to inorganic nitrate supplementation. Clinical studies have shown that inorganic nitrate treatment tends to be more effective in males, demonstrating improvements in exercise economy during submaximal effort, increased exercise capacity within the severe‐intensity domain [18], and greater reductions in blood pressure compared to females [19].

The cardiovascular effects of nitrate–nitrite–NO signaling in heart failure have been investigated in clinical trials and yielded mixed results. Lower plasma nitrite levels have been associated with increased cardiac fibrosis and hypertrophy in hypertensive patients [20]. In HFpEF patients, acute nitrate or nitrite supplementation has shown transient improvements in exercise tolerance and hemodynamics [21, 22]. However, long‐term benefits remain inconsistent, as evidenced by the INDIE‐HFpEF trial, which reported no significant improvement in exercise capacity with inhaled inorganic nitrite over four weeks [23, 24]. In HFrEF, some studies report enhanced muscle function and VO_2_ max following acute nitrate treatment [25, 26, 27], while others show neutral outcomes with longer interventions [28]. These findings underscore the importance of elucidating the underlying mechanisms and guiding clinical translation to maximize the therapeutic potential of dietary nitrate in heart failure. While HFrEF primarily affects older patients, in our study we employed young adult mice to minimize variability from age‐related comorbidities and to isolate the direct cardiac‐specific effects of dietary nitrate under controlled conditions. The MI and TAC models used are well‐established and reliably reproduce key features of HFrEF, including hemodynamic dysfunction, fibrosis, and inflammation independent of age.

### 3.2 Improved Inotropy and Diastolic Function

The inotropic effect of an inorganic enriched diet has been described previously, with short‐term treatment in healthy mice directly influencing cardiomyocytes' Ca^2+^ cycling and enhancing contractile function [29, 30, 31]. In our study, echocardiography screening showed a significant improvement in systolic function in mice with HFrEF post‐MI treated with an inorganic nitrate enriched diet. Invasive pressure–volume loop analysis in mice with HFrEF post‐TAC showed improvement in systolic parameters such as cardiac output, stroke volume, ejection fraction, and dP/dtmax. Load‐independent systolic parameters, namely end‐systolic pressure–volume relationship (ESPVR) and preload‐recruitable stroke work (PRSW), showed respectively a significant and nominal increase in the nitrate group.

Diastolic function was significantly increased only in load‐dependent conditions (dP/dTmin), while no significant changes were observed in load‐independent hemodynamic parameters obtained following inferior vena cava occlusion, such as EDPVR. This aligns with a previous study showing improved diastolic function in mice with age‐related cardiomyopathy following inorganic nitrate‐enriched diet treatment [32] Thus, other vascular factors, such as increased vascular relaxation and decreased afterload, play a major role in modulating diastolic function, promoting more efficient blood filling in a more compliant heart during diastole following inorganic nitrate‐enriched therapy.

### 3.3 Increased Vascular Relaxation

Vascular dysfunction can play a pivotal role in the progression of cardiac dysfunction and HF [33, 34]. In our HFrEF post TAC model, the inorganic nitrate treatment increased relaxation of aortic vessels, both endothelial‐dependent and independent, while it had a neutral effect on vascular contractility properties. Previous preclinical studies showed that dietary nitrate improved vascular endothelial function of coronary vessels [32] and aorta [35] in age‐related cardiovascular disease. In line with this, our data demonstrate that dietary nitrate reduced vascular stiffness leading to decreased afterload and wall stress in the left ventricle.

Previous clinical studies have demonstrated that elevation of plasma nitrite levels improved flow‐mediated dilatation (FMD) in patients with vascular dysfunction [36, 37]. This corroborates our hypothesis that boosting the noncanonical nitrate–nitrite–NO pathway in HFrEF could restore endothelial function and exert therapeutic potential.

### 3.4 Reduced Cardiac Fibrosis

Dietary nitrate reduced infarct size in mice with HFrEF post‐MI. This effect suggests potential clinical applications, where nitrate could serve as an adjunct therapy for patients with acute myocardial infarction. Previous studies showed that nitrite treatment had a cytoprotective effect during the ischemia/reperfusion model by reducing the infarct size [38], while others showed no significant changes when normalized to the area at risk in a larger cohort of patients with ST elevation myocardial infarct [39]. In preclinical experiments of cardiac ischemia reperfusion, dietary nitrate consistently reduced the infarct size [10, 40].

The timing of nitrate treatment initiation is also crucial for its antifibrotic effect. Starting the intervention too early may interfere with the early healing process, which depends on the formation of protective scar tissue in the heart. This fibrotic scaffold is essential for containing the myocardial injury and preventing further expansion of the damaged area. Our study shows that dietary nitrate supplementation was not only associated with reduced infarct size but also increased thickness of scar tissue. Thus, the value of nitrate treatment consists in preventing excessive extracellular matrix deposition that aggravates cardiac remodeling. This is an intriguing feature that has been observed in other studies that assessed the effect of therapies targeting regulatory T cells and macrophage polarization [41, 42].

Timing of intervention has been shown to be relevant also in other models of myocardial injury. Initiating inorganic nitrate treatment within the first 2 weeks of angiotensin II (AngII)‐induced injury, before manifestation of cardiac fibrosis, had significant antifibrotic effects. However, starting treatment after this period of AngII infusion appears ineffective in reversing established fibrosis due to the late intervention [20]. These findings support the potential of inorganic nitrate therapy to prevent, rather than reverse, cardiac fibrosis, and highlight the importance of early intervention following myocardial injury.

HFrEF post TAC model is characterized by increased reactive fibrosis, namely interstitial and perivascular fibrosis, predominantly driven by myofibroblast activation. The antifibrotic effect of dietary nitrate corroborates previous work showing NO‐mediated inhibition of SMAD phosphorylation and consequent nuclear traslocation [43], which primarily affects cGMP levels and PKG activity in fibroblasts [44]. In experimental models, inorganic nitrate treatment dampened sympathetic hyperactivity and oxidative stress in hypertensive rats [45, 46]. Our data showed that nitrate treatment reduced the mRNA levels of *Postn* and *Col1a1*, which are specific biomarkers of activated myofibroblasts while cardiac cGMP levels were increased. These findings reinforce the role of nitrate‐mediated NO‐cGMP signaling in suppressing fibrotic remodeling, highlighting dietary nitrate as a promising modulator of cardiac fibroblast activation and fibrosis.

### 3.5 Increased Anti‐Inflammatory Biomarkers

Inorganic nitrate‐enriched diet in HFrEF mice modulates the transcription of pro‐inflammatory and anti‐inflammatory biomarkers. In the nitrate group, expression of pro‐inflammatory cytokines IL6 and IL1b was significantly reduced in the MI model and showed a trend toward decrease in the TAC model. It is noteworthy that IL6 and IL1b could impair cardiac diastolic function by negatively affecting SERCA2a activity [47, 48]. The transcriptional levels of *Tnf* were not significantly changed between nitrate and control groups of HFrEF mice, either MI‐ or TAC‐induced. Inorganic nitrate treatment significantly increased the mRNA levels of the anti‐inflammatory cytokines such as IL10 in hearts of mice with HFrEF post MI. Macrophages M2 phenotype was predominant in both models of HFrEF treated with inorganic nitrate. This finding supports the hypothesis that NO signaling exerts an anti‐inflammatory effect and plays a crucial role in HFrEF pathogenesis by preventing the aggravation of cardiac remodeling and the expansion of the myocardial injury.

In our experimental design, dietary intervention with inorganic nitrate started at day 3 post surgery, when macrophages display increased proliferation and phagocytosis [49], and ameliorated cardiac remodeling after 4–6 weeks of treatment. This suggests that the early healing process depends on the recruitment of inflammatory cells, while the later anti‐inflammatory effects of an inorganic nitrate‐enriched diet help resolve inflammatory signaling, preventing the release of cell‐damaging substances that aggravate myocardial injury progression. Failure to resolve inflammation can result in prolonged activation of M1 macrophages, which may worsen myocardial infarction outcomes by promoting adverse cardiac remodeling [50].

Regulating inflammation in HF may help protect the vascular endothelium from damage caused by ROS and other harmful molecules generated during inflammation. This, in turn, enhances NO bioavailability, promoting vascular health and improving endothelial function.

Our findings are in line with previous studies showing that dietary inorganic nitrate treatment reduced the levels of pro‐inflammatory cytokines in the kidney following ischemia/reperfusion injury [51] and in visceral fat in a model of cardiometabolic syndrome [52]. Activation of NO signaling through overexpression of endothelial NOS had a protective effect against hepatic inflammation and insulin resistance in mice fed a high fat diet by shifting macrophage polarization toward the M2 anti‐inflammatory phenotype [53]. The anti‐inflammatory properties of dietary inorganic nitrate treatment on bone marrow‐derived macrophages were shown to be associated with a dampening of oxidative stress in obesity [52].

Moreover, inorganic nitrate ameliorated atherosclerotic disease in Apolipoprotein (Apo) E knockout (KO) mice by reducing macrophage and smooth muscle accumulation within atherosclerotic plaques, reducing systemic leukocyte rolling and adherence, and circulating neutrophil numbers that were associated with elevations of the anti‐inflammatory cytokine interleukin IL10 [54]. Treatment with IL10 attenuated inflammation and facilitated wound healing post myocardial infarct through a mechanism influenced by macrophage polarization [55]. Mice knock‐out for IL10 presented aggravated cardiac fibrosis, while IL10 treatment inhibited transdifferentiation to activated myofibroblasts in pressure overload–myocardium [56].

Our immunofluorescence staining results confirmed that dietary nitrate treatment enhances M2 macrophage polarization and reduces fibrosis activation in cardiac tissue of HFrEF mice of both MI and TAC models. Elevated serum nitrate and cardiac cGMP levels indicate preservation of the entero‐salivary pathway and support a mechanistic role for Nitrate–NO–cGMP signaling in modulating adverse cardiac remodeling. These findings highlight the therapeutic potential of targeting the nitrate–nitrite‐NO pathway in heart failure.

## 4 Conclusions

Using clinically relevant experimental models, our results demonstrated that preventive therapy with dietary inorganic nitrate supplementation in the early stage of HFrEF had four distinct beneficial effects on: (i) cardiac function, (ii) vasorelaxation, (iii) fibrosis, and (iv) inflammation. Identification of therapeutic options that are easy to deliver, safe, and acceptable to patients with HF is a current imperative.

We provided evidence that in male mice that underwent coronary artery ligation to induce MI and in mice that underwent TAC to induce pressure overload, nitrate prevented the development of HFrEF. Practical strategies for attenuating cardiovascular function deterioration following myocardial injury are limited. Implementation of clinical practice could include promoting lifestyle and dietary changes that increase nitrate intake, emphasizing vegetable‐ and fruit‐rich diets, along with the use of nitrate‐rich supplements. Inorganic nitrate represents a safe, inexpensive, and potentially powerful dietary therapeutic strategy that might help to reduce the global burden of HF.

### 4.1 Study Limitations

Our study provides robust evidence supporting the potential of dietary nitrate as a therapeutic intervention to prevent maladaptive cardiac remodeling and slow the progression of heart failure. Our findings on cardiac function, fibrosis, and inflammation offer a solid foundation for future clinical translation. However, the absence of human tissue data or patient studies limits the immediate translational applicability of these findings. We explored both ischemic and pressure overload‐induced heart failure by employing two well‐established heart failure models—myocardial infarction (MI) and transverse aortic constriction (TAC). However, only male mice were used to avoid variability from fluctuation in sex hormones and metabolism, as under various conditions, including myocardial ischemia and pressure or volume overload, female sex has been linked to reduced disease severity and improved outcomes in both clinical and preclinical studies [57, 58]. Finally, to reduce the complexity of disease development and progression, only young adult mice were used in this study. However, this may limit translatability, as heart failure is more prevalent in the elderly. Future follow‐up studies should explore the effects of dietary nitrate on heart failure across both sexes and various age groups to provide a more comprehensive understanding of the therapeutic potential of dietary nitrate.

## 5 Materials and Methods

All the material submitted is compliant with good publishing practice in physiology [59].

### 5.1 Ethics and Animal Handling

Mouse experiments were approved by the Stockholm North Ethical Committee on Animal Experiments (Ethical permits 2155‐2020 and 17816‐2021). Mouse experiments conformed to the guidelines from Directive 2010/63/EU of the European Parliament on the protection of animals used for scientific purposes. The study involving research animals complied with the Swedish Animal Welfare Act, the Swedish Welfare Ordinance, and recommendations and applicable regulations from Swedish authorities. Male C57BL/6J mice (12–16 weeks old) were purchased from Janvier Labs and were maintained on a 12‐h light/dark cycle and provided with food and water *ad libitum*.

### 5.2 HF With Reduced Ejection Fraction (HFrEF) Models

#### 5.2.1 HF With Reduced Ejection Fraction Induced by Myocardial Infarct (MI)

Male C57 BL/6J mice (12–16 weeks old) were used to induce MI and subsequent HFrEF by chronic ligation of the left anterior coronary artery. Mice were anesthetized with a gas mixture of oxygen and isoflurane (1.5%–2.0% for maintenance; up to 3% for induction). Analgesic treatment was carried out with buprenorphine (0.05 mg/kg, s.c.) 30 min before starting the surgery and for 2 days after surgery every 8–12 h. Briefly, following anesthesia with a gas mixture of oxygen and isoflurane, MI was induced by permanent ligation of the left coronary artery, as previously described [60].

#### 5.2.2 HF With Reduced Ejection Fraction Induced by Transverse Aortic Constriction (TAC)

Male mice were anesthetized with a gas mixture of oxygen and isoflurane (1.5%–2.0% for maintenance; up to 3% for induction). Analgesic treatment was carried out with buprenorphine (0.05 mg/kg, s.c.) 30 min before starting the surgery and for 2 days after surgery every 8–12 h. Briefly, Transverse Aortic Constriction (TAC) was induced in C57/BL6J mice by tying a 7–0 silk suture ligature around the aorta, against a 27‐gauge needle to yield a narrowing 0.4 mm in diameter. Then the needle is immediately removed in order to create a transverse aortic constriction (TAC) of 65%–70% to recreate a model of chronic pressure overload as described [14].

### 5.3 Animal Study Protocol and Dietary Intervention

Mice with HFrEF were fed a standard chow diet and given either regular tap water or tap water supplemented with 10 mM sodium nitrate. Consuming approximately 4 mL per day, they received around 37.5 μmol inorganic nitrate daily throughout the 4–6 weeks study. Treatment was initiated on day 3 post‐MI or TAC surgery to allow early fibrotic healing to occur without interference and to ensure adequate recovery and analgesic care for all mice prior to randomization for dietary nitrate treatment. The time of dietary nitrate intervention has been chosen with the aim of blocking the development and progression of HFrEF. Previous preclinical study showed that dietary nitrate failed to revert hypertrophy and fibrosis when the intervention started too late following myocardial injury [20]. Therefore, we selected a specific interventional time window that could produce a greater beneficial effect in both development and progression of HF. After completing in vivo cardiac characterization studies (described in detail below), the mice were euthanized through cervical dislocation and blood exsanguination. MI was confirmed by the presence of fibrotic scar tissue replacing the ischemic myocardium, while TAC was verified by the fibrotic thickening observed around the aortic arch at the site of the silk suture ligature. The hearts and other organs (e.g., aortas) were collected and processed for further analysis (described in detail below).

### 5.4 Transthoracic Echocardiography

Transthoracic echocardiography was performed 4–6 weeks post MI or TAC surgery to assess cardiac function according to standard procedures [14, 61, 62]. Mice were anesthetized with a gas mixture of oxygen and isoflurane (1.5%–2.0% for maintenance; up to 3% for induction). Echocardiography screening was performed using the Philips HDI 5000 imaging system (Philips Medical Systems, Bothell, Washington, USA), with a 7–15 MHz CL 15–7 scanning head. M‐mode imaging in the short‐axis view was used to assess left ventricular (LV) systolic and diastolic dimensions. Heart contractility was measured as % of LV fractional shortening, FS%, using the following formula: LVd − LVs/LVd × 100 where LVd and LVs represent LV diastolic and systolic dimensions respectively. EF, CO, and SV were calculated using geometric assumptions (e.g., Teichholz formula) based on LV dimensions.

### 5.5 Pressure Volume Loop Analysis

Mice with HFrEF induced by TAC, randomized for inorganic nitrate enriched or control diet, were used to perform in vivo Pressure Volume (PV) loop experiments according to standard procedures [61]. To ensure the quality and reproducibility of measurements, PV loop analysis was conducted only in the TAC model, which allows for more consistent catheter insertion via the apical route and is better suited for this type of invasive hemodynamic assessment. Briefly, the mice were anesthetized with a gas mixture of oxygen and isoflurane (2%–3%). Lung ventilation was ensured by endotracheal intubation and use of a rodent ventilator. Direct catheterization of the LV was performed by the open chest apical approach using a Millar SPR 839 catheter (size 1.4 French). Intracardiac catheterization was performed on control and nitrate‐treated HF animals to interpolate Pressure Volume curves (PV‐loops). Thereafter, inferior vena cava (IVC) occlusion was performed in order to obtain load‐independent hemodynamic parameters (i.e., end‐systolic pressure volume relationship, ESPVR). Pressure and blood volume calibration were performed before starting each procedure. For accurate measures of volume, the conductance of the heart muscle that surrounds the blood pool (parallel conductance, Vp) was calculated following hypertonic saline infusion at the end of each procedure [61]. Data were recorded and analyzed using LabChart Pro and MPVS Ultra software (AdInstruments, Sydney, New South Wales, Australia).

### 5.6 RNA Extraction and Q‐PCR for Transcriptional Biomarkers of Cardiac Remodeling

Total RNA was extracted from frozen LV biopsies using the standard TRIzol reagent (Invitrogen) protocol, and real‐time quantitative PCR (RT‐PCR) was performed on synthesized cDNA using an IQ‐5 Multicolour Real‐Time PCR Detection System (Bio‐Rad).

Specific transcriptional biomarkers were selected in order to quantify the levels of biomarkers linked to hypertrophy/wall stress: *Nppb* (NM_008726.5), *Nppa* (NM_008725.3); cardiac fibrosis: *Postn* (NM_001368678.1), *Col1a1* (NM_007742.4), *Mmp2* (NM_008610.3), *Mmp3* (NM_010809.2), *Timp1* (NM_011593.2), *Timp2* (NM_011594.3); pro‐inflammatory: *Il6* (NM_001314054.1), *Il1b* (NM_008361.4), *Tnf* (NM_013693.3), and anti‐inflammatory macrophages: *Il10* (NM_010548.2), *Cd163* (NM_001170395.1), *Mrc1* (NM_008625.2). The expression of mRNA was normalized to Gapdh (NM_001289726.1) mRNA levels. Each sample was measured in duplicate, and the average taken to represent *n* = 1. Gene expression was measured relative to internal control GAPDH, using the 2^−ΔΔCT^ algorithm and expressed as fold change over the control group.

### 5.7 Histological Analysis (Masson Trichrome Staining for Fibrosis)

Upon termination of the mice, the hearts were collected and cut transversally in two halves. The basal part of the hearts was processed in paraformaldehyde (PFA) 4% and sucrose gradient, then embedded in OCT and quickly frozen with liquid N_2_. Cryo transversal cross‐sections (10 μm thick) were stained with Masson's trichrome staining (Sigma‐Aldrich HT15‐1KT) to identify areas of connective tissue deposition (cardiac fibrosis). Images were acquired using the Zeiss Axio Scan Z1 and viewed with QuPath software. To ensure consistent histological evaluation, tissue sampling was standardized using reproducible anatomical landmarks; transverse mid‐ventricular sections were obtained at a defined level below the coronary ligation site, where infarct size is maximal, enabling reliable cross‐sectional analysis of myocardial damage and minimizing sampling bias across animals. The blue areas from Masson Trichrome staining were quantified using a manual thresholding technique in ImageJ software for measurements of infarct size normalized to the total area of the left ventricle. Perivascular and interstitial fibrosis were evaluated using an in‐house developed script compatible with Fiji/ImageJ software (version 1.38 or higher). The script was designed to perform an automated quantification of the fibrotic tissue by calculating the percentage of fibrosis in relation to the total myocardial tissue within each image. For each animal included in the analysis, an equal number of images were randomly selected from interstitial and perivascular areas at 20× magnification and averaged on a per‐mouse basis.

### 5.8 Myograph Vessels Reactivity

Thoracic aortas were dissected from HFrEF induced by TAC mice and cut into rings ~3 mm long. Rings were mounted in 10 mL organ baths containing Krebs solution heated to 37°C and bubbled with 95% O_2_ in CO_2_ in order to characterize vasoreactivity in response to various vasodilators and vasoconstrictors. Tension of aortic rings was set at 0.3 g and rings left to equilibrate for 45 min. A cumulative contraction concentration response curve to phenylephrine (10^−9^—3 × 10^−5^ M), or in phenylephrine‐pre‐contracted vessels, a relaxation curve to ACh (10^−9^—3 × 10^−7^ M) or sodium nitroprusside (SNP; 10^−9^—3 × 10^−5^ M) was then constructed. For phenylephrine contraction, the concentration was titrated until vessel tension reached EC75 of KCl maximal contraction.

### 5.9 Immunofluorescence Staining of Cardiac Cryosections

Cardiac cryo‐sections (10 μm thick) were washed 3 times with 1% PBS and incubated for 30 min at room temperature with blocking buffer (3% normal goat serum in 0.2% PBS‐T). Sections were then incubated overnight at 4°C with the following primary antibodies: CD163 (Invitrogen # 14163182, 1:100 dilution) and Periostin (AbCam #14041, 1:100 dilution). Immunoreactivity was visualized using cyanine Cy2‐ and Cy5‐conjugated antibodies (The Jackson ImmunoResearch, 1:500 dilution). Nuclei were counterstained with Hoechst (Invitrogen, 1:10 000 dilution). Images were acquired with a Leica Stellaris 5 LIA confocal microscope with a 20× objective. For each animal, an equal number of images were randomly selected from anatomically matched regions and averaged per individual. Fluorescence intensity was quantified using a custom‐developed macro compatible with Fiji/ImageJ software (version 1.38 or higher). Signal from the green and red immunofluorescence channels was measured as integrated fluorescence density and normalized to the number of nuclei per field.

### 5.10 Serum Nitrate

Serum nitrate concentrations were quantified using high‐performance liquid chromatography (HPLC) with an ENO‐20 system (EiCOM). The serum was deproteinized using ice‐cold methanol and centrifuged for 10 min at 4°C and 10 000 *g* before analyzing the samples. Briefly, nitrate was first separated via reverse‐phase ion‐exchange chromatography. The separated nitrate was then chemically reduced to nitrite using a cadmium and reduced copper reduction column. Subsequently, the resulting nitrite was derivatized using Griess reagent to form a colored diazo compound. Detection was performed via a visible light detector at 540 nm. This method provides sensitive and specific quantification of nitrate levels in serum samples.

### 5.11 Cardiac cGMP Measurement

Cardiac cGMP levels were quantified using a commercially available ELISA kit (Abcam, ab133052), following the manufacturer's instructions. To prevent cGMP degradation, the lysis buffer was supplemented with the phosphodiesterase inhibitor 3‐isobutyl‐1‐methylxanthine (IBMX, Sigma‐Aldrich, #I5879). Briefly, frozen mouse heart tissue was weighed and homogenized on ice using an IKA T10 basic tissue homogenizer with an S10N‐5G dispersing element. Tissue was homogenized in 10× volume (in μL) of 0.1 M HCl containing 10 mM IBMX. The homogenates were centrifuged, and the supernatants were used according to kit protocol. Samples were then incubated with specific anti‐cGMP antibodies in the ELISA plate wells. Optical density was measured at 405 nm with a reference correction at 580 nm using a SpectraMax microplate reader (Molecular Devices), and data were analyzed with SoftMax Pro 5.2 software. Final cGMP concentrations were normalized to the total protein content of each sample.

### 5.12 Statistical Analysis

Both data acquisition and analysis were conducted blind to treatment allocation to ensure objectivity in the results. Unpaired parametric Student's *t* test or nonparametric Kruskal–Wallis test was used to compare statistical differences between HFrEF Control and HFrEF Nitrate groups. Repeated two‐way ANOVA followed by post hoc test was used to analyze vessel myograph data. One‐way ANOVA was used to compare more than two groups. A *p* < 0.05 was considered statistically significant.

## Author Contributions

Gianluigi Pironti conceived and supervised the study. Miho Shimari, Gaia Picozzi, Ariela Boeder, Drielle Dantas Guimarães, Zhengbing Zhuge, and Gianluigi Pironti contributed to data acquisition, analysis, and/or development of methodology and provision of reagents. Jon O. Lundberg, Mattias Carlstrom, Lars H. Lund, Daniel C. Andersson, and Gianluigi Pironti contributed to data interpretation, critical revision of the manuscript, and secured funding for the study. Gianluigi Pironti drafted the original manuscript. All authors revised the manuscript for important intellectual content, approved the final version for publication, and agreed to be accountable for all aspects of the work, ensuring that any questions related to the accuracy or integrity of any part of the work are appropriately addressed.

## Conflicts of Interest

J.O.L. is co‐inventor on patents related to the therapeutic use of inorganic nitrate and nitrite. The authors declare that the research was conducted in the absence of any commercial or financial relationships that could represent a potential conflicts of interest. The other authors declare no conflicts of interest.

## Supporting information


**Figure S1:** apha70115‐sup‐0001‐Figures.docx.

## Data Availability

The data that support the findings of this study are available from the corresponding author upon reasonable request.
