# Supplementary material for: Dietary Nitrate Prevents Cardiac Dysfunction in HFrEF by Improving Hemodynamics, Ameliorating Remodeling, and Resolving Inflammation
Source: Acta Physiol (Oxf). 2025 Oct 13;241(11):e70115. doi: 10.1111/apha.70115 (PMC12516936; doi:10.1111/apha.70115)
Supplement: Supplementary file 1 — Figure S1: apha70115‐sup‐0001‐Figures.docx. [file APHA-241-e70115-s001.docx]

**Figure S1**

**Figure S1. Models of HFrEF induced by MI or TAC.**

**A.** Representative M-mode echocardiography images (short-axis view) from SHAM, MI control, and TAC control mice. **B–C.** Quantification of fractional shortening (FS%) (**B**) and ejection fraction (EF%) (**C**) demonstrates reduced systolic function in both MI and TAC groups compared to SHAM, confirming successful induction of HFrEF. MI mice display more pronounced systolic impairment than TAC mice. Data presented in scatter plot with bar showing the mean and SD of N=8-17-19 mice as indicated. Data points for MI and TAC groups correspond to those shown in Figure 1C-G and Figure S2B-H. Statistical analysis was performed using one-way ANOVA with a significance threshold of 0.05 where ‡ indicates p<0.001.

**Figure S2**

**Figure S2. Echocardiography screening in HFrEF post TAC.**

**A.** Representative M-mode echo image of one mouse from HFrEF post TAC control group and one mouse from HFrEF post TAC nitrate group. **B.** Contractility of LV, measured as fractional shortening (FS%), was significantly increased in mice with HFrEF post TAC treated with nitrate enriched diet compared to control diet. **C.** Left ventricle diastolic and systolic (**D**) dimension was significantly decreased. **E**. Interventricular septum (IVS) was nominally decreased in nitrate group. **F**. Posterior wall was not significantly changed in nitrate group. **G**. There were no significant changes in heart rate (HR) between control and nitrate group. (**H**) Ejection fraction and (**I**) cardiac output were significantly increased in the nitrate-treated group compared to TAC controls. (**J**) Stroke volume showed no significant differences between control and nitrate groups.

Data presented in scatter plot with bar showing the mean and SD of N=19-16 mice with HFrEF post TAC as indicated. Unpaired t-test was used to compare two groups (HFrEF post TAC Control and HFrEF post TAC Nitrate groups). A p < 0.05 was considered statistically significant where * indicates p<0.05; † p<0.01; ‡ p<0.001.
